# An Improved Multi-Relaxation Time Lattice Boltzmann Method for the Non-Newtonian Influence of the Yielding Fluid Flow in Cement-3D Printing

**DOI:** 10.3390/ma11112342

**Published:** 2018-11-21

**Authors:** Tiancheng Huang, Hai Gu, Jie Zhang, Bin Li, Jianhua Sun, Weiwei Wu

**Affiliations:** 1 School of Mechanical Engineering, Nantong Institute of Technology, Nantong 226002, China; njtechgy@126.com (J.Z.); njtechjxh@163.com (B.L.); njtechlyy@126.com (J.S.); 2 Jiangsu Key Laboratory of 3D Printing Equipment and Application Technology, Nantong 226002, China; 3 College of Mechanical Engineering, Yangzhou University, Yangzhou 225127, China; 006979@yzu.edu.cn

**Keywords:** Cement-3D printing, MRT-LBM, yielding fluids, forcing term, non-Newtonian effect

## Abstract

The multi-relaxation time lattice Boltzmann method (MRT-LBM) has an excellent performance in dealing with the complex flow in many different areas. According to the specific behavior of the fluids, it also has some shortcomings when applied to some special flow like as the non-Newtonian flow. In Cement-3D printing, the fluids always exhibit according to the yielding behavior. When using the standard MRT-LBM, the simulation maybe divergent. In order to solve the problem, this work presents an improved MRT-LBM considering the non-Newtonian effect as a special forcing term to ensure the stable and accurate simulation. Finally, the Poiseuille flow was used to validate the feasibility of the proposed method.

## 1. Introduction

3-D printing is an advanced technology to model parts with complex structures [1,2]. It has been widely applied in the mechanical engineering, art, bioengineering, and construction fields [3,4,5]. When it is used in the construction field, the materials are always inorganic mixtures. The fluids are typically non-Newtonian fluids [6,7,8,9]. The flow of the materials in the pipes cannot be measured easily, so simulations are required. 

In recent years, lattice Boltzmann method (LBM) has developed into a more popular method for fluid simulation. It can be applied in flow simulation with complex fluid types or various structures [10,11,12]. Although LBM has been widely applied in many areas, it is not stable or accurate in certain cases such as the non-Newtonian flow, fluids with lower or higher Reynolds number, and so on. For Newtonian fluids, the viscosity is a constant, while the viscosity of non-Newtonian fluids will change with the shearing rate. When the viscosity is close to 0 or bigger than 1, the simulation process will be unstable or inaccurate. Power-law fluids are the most common non-Newtonian fluids [13,14,15,16]. Many researchers have modified LBM for the power-law flow to achieve stable and accurate simulations. Niu et al. showed that if the relaxation time can be kept in (1/2, 1), a better stability and higher accuracy will be guaranteed [17]. Based on the above theory, Gabbanelli applied a truncated LBM in a power-law flow, which shows a better simulation [18]. Boyd conducted a local method for the power-law tunnel flow, and the results show a higher accuracy than the truncated method [19]. Nejat et al. applied a second-order lattice Boltzmann method in a power-law flow simulation [20]. Orestis et al. presented a local lattice Boltzmann method for power-law fluids to avoid divergence. It should be noted that the power-index should be kept in a certain range to ensure the validity [21]. In addition to the power-law flow, some other yielding fluids have also been studied. Tang et al. incorporated the Papanastasiou exponential improved method to simulate Bingham flow [22]. Chen Songgui et al. used MRT-LBM to study Bingham flow, which obtains stable simulations [23]. Wu Weiwei et al. proposed a truncated method for Herschel–Bulkley fluids by using LBM and MRT-LBM, and the effectiveness has been validated by theoretical solutions of Poiseuille flow [24,25]. In Cement-3D printing, the cement paste represents the yielding fluids characteristic, which belongs to Herschel–Bulkley fluids.

Compared with power-law fluids, the researches on yielding fluids are insufficient. Although some researchers proposed improved methods for Bingham fluids, they are not well suited for Herschel–Bulkley fluids. The power-index has an important impact on the simulation. In this work, an improved method was proposed for substituting the non-Newtonian influence of a Herschel–Bulkley fluid into external forcing term. Then cement paste flow in 3-D printing was numerically simulated using the proposed method. Finally, the improved method was validated using a Poiseuille flow. 

## 2. Improved MRT-LBM for Yielding Fluids

### 2.1. Rheological Equation of Yielding Fluids

Common yielding fluids mainly include Bingham fluids and Herschel–Bulkley fluids. They can be expressed using a universal equation:

(1) $$\left\{ \begin{array}{ll} \dot{\gamma }=0, & \left|\tau_{a}\right|<\tau_{0}\\ \tau_{a}=\tau_{0}+K{\dot{\gamma }}^{n}, & \left|\tau_{a}\right|\geq \tau_{0} \end{array}\right.$$

where $\dot{\gamma }$ is the shearing rate, *τ_a_* is the shearing stress, *K* is the viscosity coefficient, *τ*_0_ is the initial yielding stress, and *n* is the power-law index. When *n* = 1, the fluids will be Bingham fluids; otherwise, it corresponds to Herschel–Bulkley fluids. The kinematic viscosity *ν* can be calculated using:

(2) $$\left\{ \begin{array}{ll} \dot{\gamma }=0, & \left|\tau_{a}\right|<\tau_{0}\\ \nu =\frac{\tau_{0}}{\left|\dot{\gamma }\right|}+K{\left|\dot{\gamma }\right|}^{n-1}, & \left|\tau_{a}\right|\geq \tau_{0} \end{array}\right.$$

In 1997, Papanastasiou and Boudouvis proposed a modified equation to simplify the constitutive model [26]:

(3) $$\nu_{ap}=K{\left|\dot{\gamma }\right|}^{n-1}+\frac{\tau_{0}}{\rho \left|\dot{\gamma }\right|}\left[1-\exp(-m\left|\dot{\gamma }\right|)\right]$$

where *ρ* is the density, and *m* is used to control the increase of stress. When *m* approaches zero, the equation will become the constitutive model of power-law fluids.

### 2.2. Improved MRT-LBM

Standard MRT-LBM in two-dimensional space (D2Q9 model) with a forcing term can be expressed as [27]:

(4) $$f(r+e_{i}\delta t,t+\delta t)-f(r,t)=-M^{-1}\bar{S}M\left[f(r,t)-f^{eq}(r,t)\right]+\delta tF^{\prime }$$

where ***r*** is the displacement vector, ***e_i_*** is the discrete velocity, ***F′*** is an external force, and *δt* is the time step size, which is always set as *δt* = 1. The second item on the right side relates to the external force.

***f*** is a distribution function and ***f^eq^*** is an equilibrium distribution function, which is defined as:

(5) $$f^{eq}(r,t)=\omega_{i}\rho \left[1+\frac{e_{i}\cdot u}{c_{s}^{2}}+\frac{{\left(e_{i}\cdot u\right)}^{2}}{2c_{s}^{4}}-\frac{u^{2}}{2c_{s}^{2}}\right]$$

where *c_s_* represents the lattice sound speed, where $c_{s}^{2}=c^{2}/3$; ***u*** is the velocity vector; *ω_i_* is the weight coefficient, given by *ω*_0_ = 4/9, *ω_i_* = 1/9 for *i* = 1–4, and *ω_i_* = 1/36 for *i* = 5–8. ***M*** is a transformation matrix:

(6) $$M=\left[\begin{array}{ccccccccc} 1 & 1 & 1 & 1 & 1 & 1 & 1 & 1 & 1\\ -4 & -1 & -1 & -1 & -1 & 2 & 2 & 2 & 2\\ 4 & -2 & -2 & -2 & -2 & 1 & 1 & 1 & 1\\ 0 & 1 & 0 & -1 & 0 & 1 & -1 & -1 & 1\\ 0 & -2 & 0 & 2 & 0 & 1 & -1 & -1 & 1\\ 0 & 0 & 1 & 0 & 1 & 1 & 1 & -1 & -1\\ 0 & 0 & -2 & 0 & -2 & 1 & 1 & -1 & -1\\ 0 & 1 & -1 & 1 & -1 & 0 & 0 & 0 & 0\\ 0 & 0 & 0 & 0 & 0 & 1 & -1 & 1 & -1 \end{array}\right]$$

The discrete velocity ***e_i_*** is expressed as:

(7) $$e_{i}=\left\{ \begin{array}{ll} (0,0), & i=0\\ c(\cos\frac{i-1}{2}\pi ,\sin\frac{i-1}{2}\pi ), & i=1,2,3,4\\ \sqrt{2}c(\cos\frac{2i-9}{4}\pi ,\sin\frac{2i-9}{4}\pi ), & i=5,6,7,8 \end{array}\right.$$

The lattice speed *c* = *δx*/*δt*, *δx* represents the lattice step size, whose value is always set as 1.

The principal diagonal symmetric matrix $\bar{S}$ mainly has an important effect on the relaxation process. The specific expression is given by:

(8) $$\bar{S}=diag(s_{0},s_{1},s_{2},s_{3},s_{4},s_{5},s_{6},s_{7},s_{8})$$

where *s*_0_, *s*_3_, *s*_5_, *s*_7_, and *s*_8_ are common relaxation factors, *s*_3_ and *s*_5_ have the same meaning, and *s*_7_ and *s*_8_ have the same meaning. *s*_1_ is related to the viscosity, and *s*_2_ can be used to adjust the stability of numerical simulation. *s*_4_ and *s*_6_ can be used to improve the accuracy of MRT-LBM. The factors have the following relations: *s*_3_ = *s*_5_, where they have no influence on macroscopic process; *s*_4_ = *s*_6_, where they affect the accuracy of MRT model; and *s*_7_ = *s*_8_, where they represent relaxation factors. Then, *s*_0_ is an important related factor to the density, and it also has no effect on the macroscopic process. *s*_1_ is usually related to viscosity. The stability can also be improved by adjusting it. The factor *s*_2_ also has an impact on the stability. Here, *s*_1_ and *s*_2_ are set to be 0.8. According to the above description, *s*_0_, *s*_3_, and *s*_5_ are selected arbitrarily as 0. *s*_4_ and *s*_6_ are equal to 1.9. *s*_7_ and *s*_8_ will be discussed further below.

With MRT-LBM, kinematic viscosity is defined as:

(9) $$\nu =c_{s}^{2}\left(\frac{1}{s_{8}}-\frac{1}{2}\right)\delta t$$

For a standard single relaxation time LBM (LBM mentioned below refers to a standard single relaxation time LBM), the strain rate tensor is described as:

(10) $$S_{\alpha \beta }=-\frac{1}{2\rho \tau c_{s}^{2}}\sum_{i=0}^{8}e_{i\alpha }e_{i\beta }\left(f_{i}-f_{i}^{eq}\right)$$

where *ρ* is the density, and *τ* is the relaxation time, where is *τ* = 1/*s*_8_. However, the strain rate tensor in standard MRT-LBM is different from LBM, which is expressed as:

(11) $$S_{\alpha \beta }=-\frac{1}{2\rho c_{s}^{2}\delta t}\left[\sum_{i=0}^{8}e_{i\alpha }e_{i\beta }\sum_{j=0}^{8}{\left(M^{-1}\bar{S}M\right)}_{ij}\left(f_{j}-f_{j}^{eq}\right)\right]$$

Then, the second invariant of strain rate tensor can be acquired using Equation (11) as:

(12) $$D_{II}=\sum_{\alpha ,\beta =1}^{l}S_{\alpha \beta }S_{\alpha \beta }$$

With Equation (12), the shearing rate can be calculated as:

(13) $$\dot{\gamma }=\sqrt{2D_{II}}$$

According to the equality of two different expressions in Reference [21], then substituting viscosity into the related equation, the stress tensor *σ_αβ_* can be described as:

(14) $$\sigma_{\alpha \beta }=-P\delta_{\alpha \beta }+2KS_{\alpha \beta }+\left\{2K\left[{\left|\dot{\gamma }\right|}^{n-1}-1\right]+\frac{\tau_{0}}{\left|\dot{\gamma }\right|}\left[1-\exp\left(-m\left|\dot{\gamma }\right|\right)\right]\right\}S_{\alpha \beta }\;$$

where *δ_αβ_* is a Kronecker delta, *K* is the viscosity coefficient, and *P* is the pressure. In the following section, the forcing term will be used to explain the non-Newtonian effect. For standard MRT-LBM with external force, the forcing term is expressed as [23]:

(15) $$F^{\prime }=M^{-1}\left(I-\frac{\bar{S}}{2}\right)M\bar{F}$$

Further, the item $\bar{F}$ in Equation (15) can be calculated using:

(16) $${\bar{F}}_{i}=\omega_{i}\left[\frac{e_{i}\cdot F}{c_{s}^{2}}+\frac{\left(uF+Fu):(e_{i}e_{i}-c_{s}^{2}I\right)}{2c_{s}^{4}}\right]$$

The Navier–Stokes equation would be expanded by Chapman–Enskog at incompressible limit as:

(17) $$\rho \partial_{t}(u_{\beta })+(\rho u_{\alpha })\partial_{\alpha }u_{\beta }=-\partial_{\beta }P+2\mu \partial_{\alpha }S_{\alpha \beta }$$

where *u_α_* and *u_β_* are the components of *u*, and *μ* is the dynamic viscosity. The relation between kinematic viscosity and dynamic viscosity is *ν* = *μ*/*ρ*. Also, the above equation can also be described using the following equation, which includes the forcing term:

(18) $$\rho \partial_{t}\left(u_{\beta }\right)+\left(\rho u_{\alpha }\right)\partial_{\alpha }u_{\beta }=-\partial_{\beta }P+2K\partial_{\alpha }S_{\alpha \beta }+F$$

Combining Equations (3) and (14), with Equation (17), the force related term ***F*** in Equation (18) can be obtained as:

(19) $$F_{i}=\left\{2\mu_{0}\left[{\left(\sqrt{2D_{II}}\right)}^{n-1}-1\right]+\frac{\tau_{0}}{\left(\sqrt{2D_{II}}\right)}\left[1-\exp\left(-m\sqrt{2D_{II}}\right)\right]\right\}\partial_{\alpha }S_{\alpha \beta }$$

Thus, the non-Newtonian effect described as a special external force will be achieved. Combining the shearing rate obtained from Equation (13) with Equation (3), the parameter *s*_8_ for next iteration could be calculated. The specific programming process based on software MATLAB 2014a (MathWorks, Natick, MA, USA) can be conducted according to the following steps:

(1) Conduct the physical transformation according to the dimension theory. The Reynolds number is taken as the key criteria, and then the other parameters used in simulation can be obtained.
(2) Set the simulation domain and initial conditions, especially the initial value of the factor *s_8_*.
(3) Calculate the equilibrium distribution function according to Equation (5).
(4) Conduct the collision step, where the specific function is shown as:
  (20) $$f^{+}(r,t)=f(r,t)+M^{-1}\bar{S}[\varrho (r,t)-\varrho^{\mathrm{eq}}(r,t)]+\delta F^{\prime }$$
  where *ϱ*(***r***,*t*) = ***Mf***(***r***,*t*), *ϱ^eq^*(***r***,*t*) = ***Mf^eq^***(***r***,*t*), and the force term is mainly used to explain the non-Newtonian effect of yielding fluids. The calculations can be seen in Equations (15), (16), and (19).
(5) Calculate the strain rate tensor and shearing rate by using Equations (11)–(13), then the kinetic viscosity can be obtained using Equation (3) and the relaxation factor *s*_8_ will be updated.
(6) Conduct the streaming step, where the streaming function is shown as:
  (21) $$f(r+e_{i}\delta t,t+\delta t)=f^{+}(r,t)$$
(7) Process the boundary condition, where the non-equilibrium bounce-back scheme is taken as the boundary method.
(8) Conduct the next calculation from Step (3).
(9) Calculate the density and velocity, where the corresponding equations are:
  (22) $$\rho =\sum_{i}f_{i}(r,t),\rho u=\sum_{i}e_{i}f_{i}(r,t)+\frac{\delta t}{2}F^{\prime }$$

## 3. Flow Analysis for the Mixture Fluids in Cement-3D Printing

### 3.1. Rheological Equation of Fluids

Many experiments have been done to determine the detailed types and proportions of materials. The specific components are ordinary Portland cement, water, polycarboxylate water reducing agent, modified rod soil, and cellulose ether. The corresponding proportion were 1:0.4:1.5‰:0.4‰:1.2‰. The rheological equation for mixture fluids can be obtained using a viscometer, which is described as:

(23) $$\left\{ \begin{array}{ll} \tau_{a}=3.899+1.103{\dot{\gamma }}^{0.633} & ,\left|\tau_{a}\right|>3.899\\ \dot{\gamma }=0 & ,\left|\tau_{a}\right|<3.899 \end{array}\right.$$

With Equation (23), it can be found that the mixture fluids were Herschel–Bulkley fluids. It belongs to typical yielding non-Newtonian fluids.

### 3.2. Structure of Extruding Head

**Figure 1:**
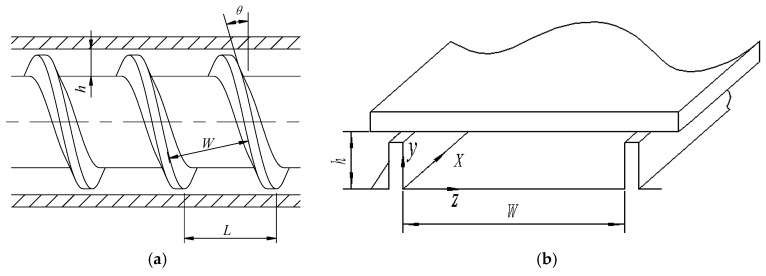
Structure of the extruder: (**a**) diagram of the extruder, and (**b**) spreading structure of the extruder.

In the Cement-3D printing experiment, the structure of the printing head is the key technology. According to repeated testing, the single screw extruder was taken as the printing head. The detailed structure is shown as Figure 1a. When expanded along a spiral, a cavity can be obtained, as seen in Figure 1b. 

The specific meanings and values of the key parameters have been listed in Table 1. 

### 3.3. Flow Simulation using Improved MRT-LBM

**Figure 2:**
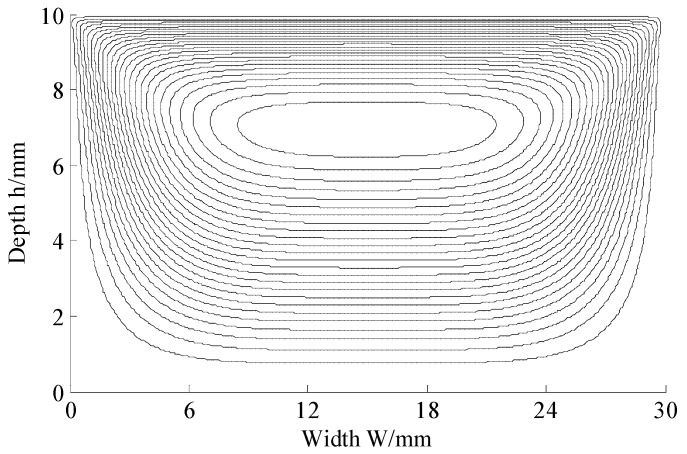
Streamlines figure.

The lattice number was set as 480 × 160. The rotation speed of the screw was 40 r/min. The diameter of the screw was designed to be 50 mm. The velocity was only set at the upper surface in Figure 1b and the velocities at other three surfaces were 0. With the above description, flow simulations were conducted. A streamlines figure could be obtained as seen in Figure 2. The simulation of the paste flow using standard MRT-LBM was also conducted, while the result was divergent. It was mainly caused by the non-Newtonian effect.

**Figure 3:**
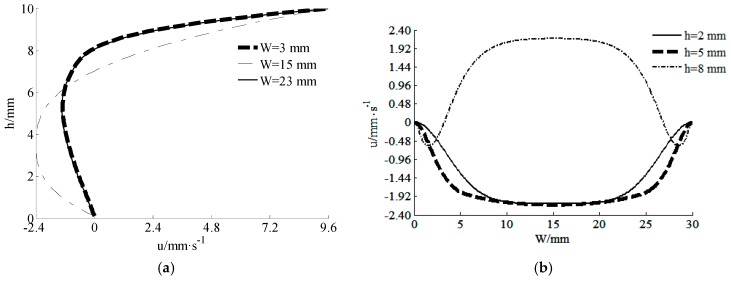
Distributions of the velocity component *u* at different directions: (**a**) velocity *u*-depth *h*, and (**b**) width *W*-velocity *u*.

In addition, velocity components were also analyzed. The velocity distributions of the component *u* at different directions are given in Figure 3. In Figure 3a, it can be found that the biggest velocity *u* was near the inner wall of the barrel and the smallest velocity *u* was near the outer wall of the screw. In Figure 3b, it shows that the biggest velocity *u* was near the center line of the channel and the smallest velocity *u* was at the wall of swirling leaves.

**Figure 4:**
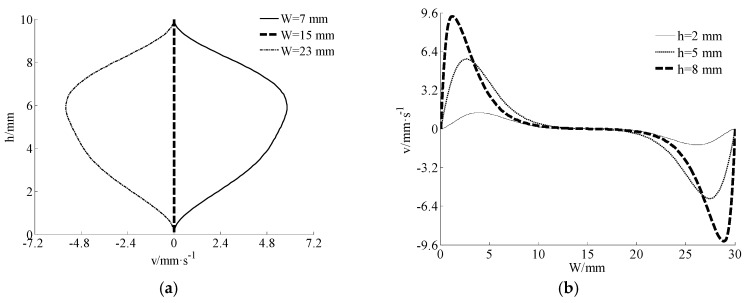
Distributions of the velocity component *v* at different directions: (**a**) velocity *v*-depth *h*, and (**b**) width *W*-velocity *v*.

It can be found from Figure 4a that the biggest velocity *v* was at the midway between the barrel and the screw. Then the velocity *v* was always 0 along the center line of channel. In Figure 4b, the velocity *v* near the outer wall of the screw was approximately 0 and it fluctuated near the wall of the swirling leaves. It was bigger when the position was closer to the inner wall of the barrel.

## 4. Validation and Discussion

The obtained results demonstrate the stability of the new, improved MRT-LBM. To ensure accuracy, we took the theoretical results of Poiseuille flow to compare with numerical results of the proposed method. The theoretical solution of yielding fluids is shown as:

(24) $$u(y)=\left\{ \begin{array}{ll} \frac{n}{n+1}{\left(-\frac{1}{\mu_{0}}\frac{\partial P}{\partial x}\right)}^{1/n}\left[{\left(\frac{H}{2}+\frac{\tau_{0}}{\partial P/\partial x}\right)}^{(n+1)/n}-{\left(y_{\tau }+\frac{\tau_{0}}{\partial P/\partial x}\right)}^{(n+1)/n}\right] & ,0\leq \left|y\right|\leq y_{\tau }\\ \frac{n}{n+1}{\left(-\frac{1}{\mu_{0}}\frac{\partial P}{\partial x}\right)}^{1/n}\left[{\left(\frac{H}{2}+\frac{\tau_{0}}{\partial P/\partial x}\right)}^{(n+1)/n}-{\left(y+\frac{\tau_{0}}{\partial P/\partial x}\right)}^{(n+1)/n}\right] & ,y_{\tau }\leq \left|y\right|\leq \frac{H}{2} \end{array}\right.$$

where *H* represents the distance between the two parallel plates, and *y_τ_* is a critical point, which is equal to:

(25) $$y_{\tau }=-\tau_{0}/\left(\partial P/\partial x\right)$$

We consider that the pivotal factors mainly included initial yielding stress, power-index, and lattice number. The accuracy will be discussed using the result of the relative error. The equation of the error *e_r_* is:

(26) $$e_{r}=\sum_{i=1}^{N}{\left(1-\nu_{i}^{numerical}/\nu_{i}^{theoretical}\right)}^{2}$$

The power index *n* was set as 0.5, 1, 2, where these values correspond to shear-thinning fluids, Bingham fluids, and shear-thickening fluids, respectively. For different power indexes, the effect of lattice numbers and initial yielding stresses were explored. To avoid the effect of other factors, we set the pressure gradient ∂P/∂x = −1.0 × 10^−5^, viscosity coefficient $\mu_{0}$ = 0.01, density *ρ* = 1, and distance *H* = 1. Lattice numbers were set in the range of 50–250 and the initial yielding stresses were selected as 1.0 × 10^−6^, 2.5 × 10^−6^, and 3.5 × 10^−6^.

**Figure 5:**
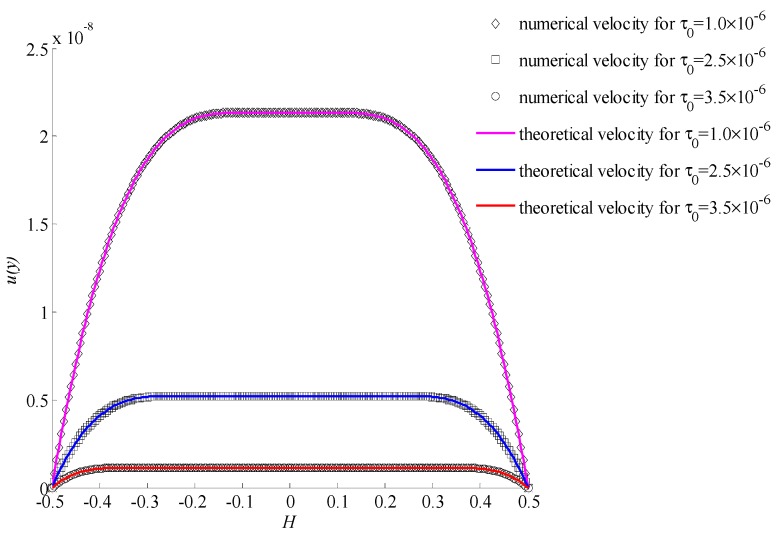
The velocities for *n* = 0.5 with different initial yielding stresses.

**Figure 6:**
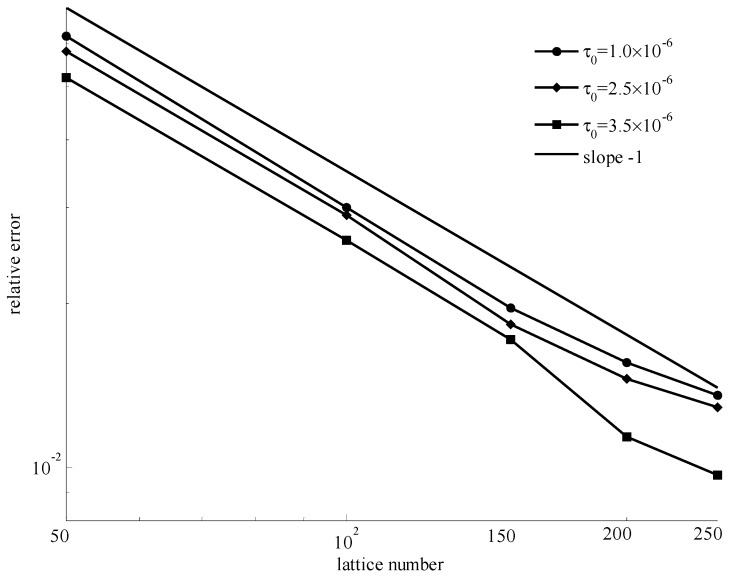
Relative errors for *n* = 0.5 with different initial yielding stresses and lattice numbers.

In Figure 5, it shows the numerical velocities and theoretical velocities for different initial yielding stresses when the power index *n* = 0.5 and lattice number was 200. It can be seen that the numerical velocities were consistent with the theoretical velocities at different initial yielding stresses. In Figure 6, it gives the relative error for each case of three initial yielding stresses with different lattice numbers. The relative error decreased with increasing lattice number.

**Figure 7:**
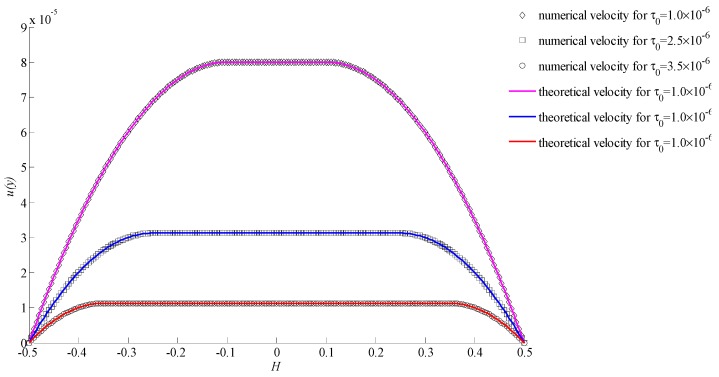
Velocities for *n* = 1 with different initial yielding stresses.

**Figure 8:**
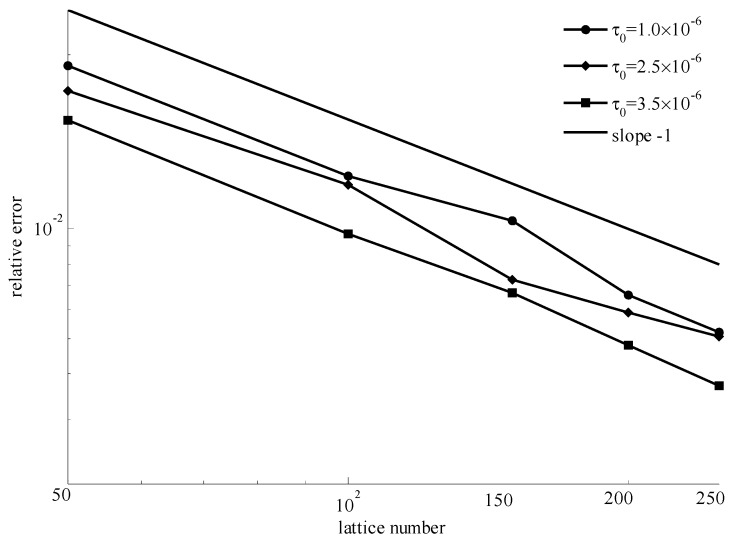
Relative errors for *n* = 1 with different initial yielding stresses and lattice numbers.

When the power index *n* = 1, it corresponds to a special kind of yielding fluids named Bingham fluids, and the velocities of the simulation and theory at three different initial yielding stresses are shown in Figure 7. It shows that the numerical results also had a good consistency with the theoretical results for all cases. The figure of relative errors are given in Figure 8 for three situations. It suggests that the bigger the lattice number, the smaller the relative error will be.

**Figure 9:**
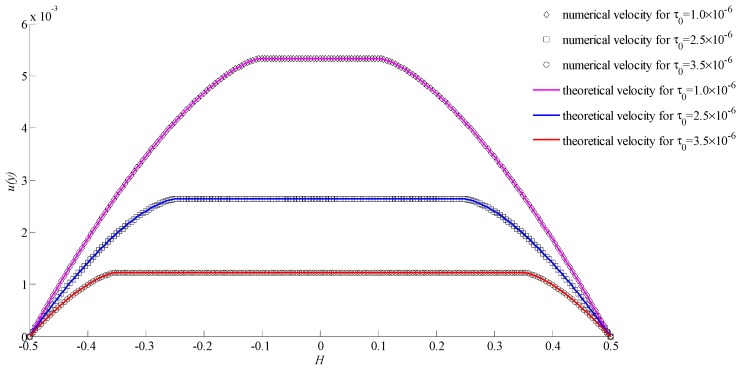
Velocities for *n* = 2 with different initial yielding stresses.

**Figure 10:**
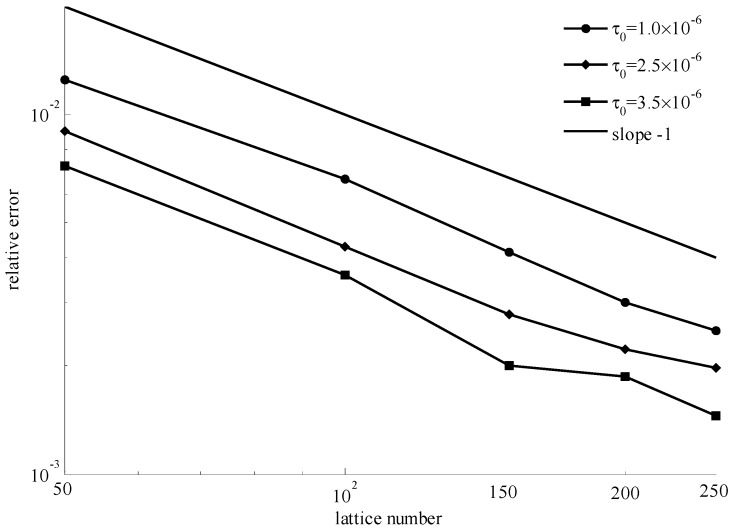
Relative errors for *n* = 2 with different initial yielding stresses and lattice numbers.

When the power index *n* = 2, it belongs to shear-thickening fluids. The velocities of simulation and theory at three different initial yielding stresses are shown in Figure 9. In Figure 10, it gives the tendency between relative error and lattice number. Both the good consistencies and small relative errors demonstrate that the proposed method is effective.

In Figure 5, Figure 7, and Figure 9, the only difference of conditions is the power index. It was found that the power index had an important effect on velocity. With the increasing of the power index, the velocity increased rapidly. Also, the initial yielding stress affected the velocity significantly. The smaller initial yielding stress led to a bigger velocity. For different initial yielding stresses and power indexes, all results showed a good consistency. This indicates that the proposed method is stable and valid.

In Figure 6, Figure 8, and Figure 10, for different power indexes and initial yielding stresses, the similarity was that the relative error decreased with increasing lattice number. The slope of each line was very close to −1. The smaller errors indicated that the improved method is accurate.

## 5. Conclusions

The non-Newtonian effect will result in a poor stability and accuracy when using standard LBM in the numerical simulation of yielding fluids. To improve the situation, a modified multi-relaxation-time lattice Boltzmann method was proposed. The non-Newtonian part was modeled with a special forcing term. Then the improved method was applied in a simulation of mixed paste in the extruder of Cement-3D printing. Stable streamlines and velocity distributions were obtained. For the velocity component *u*, the biggest velocity in two directions occurred at the position of the inner wall of the barrel and the center of the channel, respectively. The smallest velocity in two directions occurs at the position of the outer wall of screw and the wall of swirling leaves, respectively. For the velocity component *v*, it was 0 when the position was at the center of the channel and the outer wall of the screw. In order to prove the stability and accuracy of the method, numerical solutions of the Poiseuille flow were compared with theoretical solutions. The numerical results were consistent with the theoretical results. For the cases of different initial yielding stresses and power-indexes, the relative errors were small enough to verify the feasibility of the improved method.

## Figures and Tables

**Table 1:** The key parameters of the extruder.

Factor	Meaning	Value
*W*	the width of the channel	30 mm
*h*	the depth of the channel	10 mm
*θ*	angle of lead	20°
